# Outcome after proton beam therapy versus photon-based radiation therapy in childhood-onset craniopharyngioma patients—results of KRANIOPHARYNGEOM 2007

**DOI:** 10.3389/fonc.2023.1180993

**Published:** 2023-10-27

**Authors:** Carsten Friedrich, Svenja Boekhoff, Martin Bischoff, Julia Beckhaus, Panjarat Sowithayasakul, Gabriele Calaminus, Maria Eveslage, Chiara Valentini, Brigitte Bison, Semi B. Harrabi, Mechthild Krause, Beate Timmermann, Hermann L. Müller

**Affiliations:** ^1^ Department of Pediatrics and Pediatric Hematology/Oncology, University Children’s Hospital, Carl von Ossietzky University Oldenburg, Klinikum Oldenburg AöR, Oldenburg, Germany; ^2^ Department of Particle Therapy, University Hospital Essen, West German Proton Therapy Centre Essen (WPE), West German Cancer Center (WTZ), Essen, Germany; ^3^ Department of Pediatrics, Faculty of Medicine, Srinakharinwirot University, Bangkok, Thailand; ^4^ Department of Pediatric Hematology/Oncology, University of Bonn Medical Center, Bonn, Germany; ^5^ Institute of Biostatistics and Clinical Research, University of Münster, Münster, Germany; ^6^ Department of Radiotherapy and Radiation Oncology, Faculty of Medicine and University Hospital Carl Gustav Carus, Technische Universität Dresden, Dresden, Germany; ^7^ Diagnostic and Interventional Neuroradiology, Faculty of Medicine, University of Augsburg, Augsburg, Germany; ^8^ Department of Radiation Oncology, Heidelberg University Hospital, Heidelberg, Germany; ^9^ National Center for Tumor Diseases (NCT) Dresden with German Cancer Research Center (DKFZ), University Hospital and Faculty of Medicine Dresden, Helmholtz-Zentrum Dresden – Rossendorf, Dresden, Germany; ^10^ German Cancer Consortium (DKTK), Dresden and German Cancer Research Center (DKFZ) Heidelberg, Dresden, Germany; ^11^ Department of Particle Therapy, University Hospital Essen, West German Proton Therapy Centre Essen (WPE), West German Cancer Centre (WTZ) and German Cancer Consortium (DKTK), Essen, Germany

**Keywords:** craniopharyngioma, proton beam therapy, irradiation, quality of life, hypothalamus, obesity, photon-based radiation therapy, survival

## Abstract

**Background:**

Proton beam therapy (PBT) is being increas16ingly used to treat residual craniopharyngioma (CP) after hypothalamus-sparing surgery. Compared to photon-based radiation therapy (XRT) with PBT, less irradiation in the penumbra reduces the scattered dose to critical organs neighboring but outside the area of treatment, minimizing the risk of sequelae.

**Patients and methods:**

Between 2007 and 2019, 99 of 290 (34%) childhood-onset CP patients recruited in KRANIOPHARYNGEOM 2007 received external radiation therapy (RT) (65% PBT, 35% XRT). Outcome was analyzed in terms of survival, endocrinological and anthropometric parameters (BMI and height SDS), quality of life (QoL using PEDQOL), and functional capacity (FMH) with special regard to irradiation technique.

**Results:**

PBT became predominant (used in 43% and 72% of all irradiated patients registered within the first and second halves of the recruitment period, between 2008 and 2013 and 2013 and 2018, respectively). Five-year event-free survival rates after PBT or XRT were comparable (92% ± 4% vs. 91% ± 4%, *p* = 0.42) and higher than for the whole cohort since diagnosis, including non-RT patients (37% ± 4%). Radiation doses to the hypothalamus and pituitary did not differ between PBT and XRT. Endocrine deficits due to disturbances of the hypothalamic-pituitary axis (HPA) were already common before irradiation. During the first 5 years after CP diagnosis/RT, no differences between PBT, XRT, and non-RT CP patients concerning functional capacity and anthropometric parameters have been obtained. Only for the PEDQOL domain “physical function”, parental-assessed QoL was lower 12 months after PBT versus XRT or non-RT patients.

**Conclusion:**

QoL, functional capacity, degree of obesity, and endocrinopathy varied over time from diagnosis, but by 5 years, there was no significant difference between PBT and XRT upfront or delayed, nor was there any compromise in historic survival rates, which remained high >90%. RT of any type is extremely effective at stabilizing disease after hypothalamic-sparing surgery. The purported specific benefits of PBT-reducing sequelae are not proven in this study where the organ of critical interest is itself diseased, increasing an urgent need to better address and treat the tumor-induced endocrine harm from diagnosis in dedicated pituitary services. Other hypothesized benefits of PBT versus XRT on vascular events and secondary cancers await longer comparison.

**Clinical trial registration number:**

https://clinicaltrials.gov/study/, identifier NCT01272622.

## Introduction

Although craniopharyngiomas (CP) are slow-growing WHO °I tumors of the sellar and parasellar region (1), they may infiltrate adjacent structures, causing relevant morbidity and mortality (2). Previous studies show that attempts at gross-total resection (GTR) in patients with tumors invading the hypothalamus can result in significant morbidity in terms of hypothalamic dysfunction and altered neurophysiological and neuropsychological profiles (3–7). KRANIOPHARYNGEOM 2007 is a prospective multicenter trial in children and adolescents diagnosed with CP at an age younger than 18 years and treated with a risk-adapted multimodality strategy using a preoperative radiological grading system to avoid significant morbidity (8). In hypothalamic involvement grade 0/1 (no involvement of anterior hypothalamic structures in front of mammillary bodies on MRI), GTR is recommended, whereas in hypothalamic involvement grade 2 (involvement of posterior hypothalamic nuclei including mammillary bodies and structures beyond these), a subtotal tumor resection (STR) is aimed to keep the integrity of hypothalamic structures (9–12).

Incomplete surgery is associated with CP progression in 50%–91%, leading to their eligibility of these patients for postoperative radiation therapy (RT) if repeated surgery should be avoided (13). The optimal timing for RT after STR with a significant residual tumor remains an open question. In KRANIOPHARYNGEOM 2007, a subgroup of patients aged >5 years at diagnosis with postoperative residual tumors were randomized for immediate RT or RT at progression, showing no meaningful differences in quality of life (QoL) (8). We reported that for the whole cohort, RT was very useful in preventing further progression after STR, and the hypothalamus-sparing treatment strategy was associated with a higher long-term QoL (8).

Different RT techniques were used for the adjuvant treatment of CP in the KRANIOPHARYNGEOM 2007 trial. The advantage of proton beam therapy (PBT) is the lower dose to critical organs, as shown by treatment planning studies, which may decrease the risk of neurocognitive, vascular, and optic nerve complications and second cancers (14–16). However, studies comparing PBT and XRT in one large patient cohort are scarce.

Herein, we analyzed the use of different RT techniques within the KRANIOPHARYNGEOM 2007 cohort and whether PBT compared to XRT or no adjuvant RT influenced outcome parameters for survival and hypothalamic syndrome.

## Materials and methods

### Patients

Between 2007 and 2019, 290 patients diagnosed with childhood-onset adamantinomatous CP at an age ≤18 years have been enrolled in the trial KRANIOPHARYNGEOM 2007 (NCT01272622). The surgical approach was not part of the KRANIOPHARYNGEOM 2007 trial, but a hypothalamic sparing technique according to the preoperative radiological grading was recommended (8). Confirmation of the histological diagnosis adamantinomatous CP by pathological reference assessment was mandatory. Data on histological and molecular tumor characteristics, treatment, early and late toxicities, and tumor control was prospectively collected. For this cross-sectional study, trial data on physical condition, anthropometric parameters, clinical manifestations, imaging results, resection status, and RT (technique, dosage, age at initiation) has been analyzed. Endocrine deficiencies were assessed preoperative, 6 months postoperative, and then once yearly and interpreted by the local endocrinologist using local center reference values.

### Neuroimaging

Cranial MRIs were performed at the time of CP diagnosis and after initial diagnosis/progression prospectively every 3 months in the first year and then every 6 to 12 months for at least 10 years of follow-up using the guidelines of the KRANIOPHARYNGEOM 2007 protocol (8). A central imaging review by an experienced neuroradiologist (B.B.) blinded for clinical and surgical information included assessment of CP location, preoperative hypothalamic involvement (HI), surgical hypothalamic lesions (HL), and postoperative residual tumor on pre- and postoperative axial, coronal, and sagittal planes of magnetic resonance imaging (MRI). CP location was classified according to the degree of HI: grade 0, no HI; HI grade 1, involving anterior hypothalamic structures and not mammillary bodies and hypothalamic structures dorsal of those; HI grade 2, involving both anterior + posterior hypothalamic areas, i.e., mammillary bodies and areas dorsal of those (8, 10, 17). Surgical HLs were graded according to the same criteria in three categories: HL grade 0, no HL; HL grade 1, anterior HL; and HL grade 2, anterior + posterior HL. The tumor size calculation of CP was performed using the formula “½ (A × B × C)” (aligned to the ellipsoid model: 4/3 π [A/2 × B/2 × C/2]), with A, B, and C being the maximum dimensions in the standard planes: axial (transverse, A), coronal (cranio-caudal, B), and sagittal (antero-posterior, C). A complete resection needed to be confirmed by centrally reviewed MRI.

### Radiotherapy

RT was performed between 3 and 12 weeks postoperatively, or as soon as possible after the diagnosis of recurrence if no repeat surgery was performed. All patients were treated once a day, five times a week, with a fraction of 1.8 Gy per session. The total dose was 54.0 Gy. In general, CT-based therapy planning was recommended, allowing the evaluation of the integral dose within the target volumes and organs at risk. The clinical target volume corresponded to the areas of preoperative tumor contact adjusted for current anatomy, including the residual tumor extent shown on postoperative imaging, with a safety margin of 0.5 cm. The planning target volume captured the clinical target volume depending on the geometric precision of the technique used, typically 0.3–0.5 mm. The following organs at risk should have been contoured to create the integral dose distribution: brainstem, chiasm, pituitary gland, optic nerve, bulbus, lens, thalamus, hypothalamus, and inner ear. The planning target volume and the organs at risk were defined by image fusion with a diagnostic MRI not older than 21 days before the CT-based planning. The three-dimensional conformation technique with standing fields was commonly applied. A convergence technique was also used if the clinical target volume was spherical in configuration. In this situation, the dose specification was based on the specifications for stereotactic one-time therapy. The dose specifications were based on the ICRU 50/62 report. If possible, the target volume should be irradiated within the tolerance ranges of 95%–107%. The dose maximum and minimum within the target volume as well as possible dose peaks “hot spots” (dose maximum outside the target volume) were specified.

### Anthropometric parameters

Standing height was measured in triplicate, of which the median was calculated as height SDS according to the Prader et al. references (18). Patients were weighed on calibrated electronic step-scales. The degree of obesity was derived from the body mass index (BMI) SDS (*w*/*h* ^2^; *w* = weight (kg), *h* = height (m)) according to the reference of Rolland-Cachera et al. (19).

### Quality-of-life questionnaire

In CP patients diagnosed at an age ≥5 years, health-related QoL was analyzed using the Pediatric Quality of Life (PEDQOL) (8, 20) questionnaire at time points 1, 3, and 5 years after CP diagnosis/RT. Furthermore, parental estimation of their child’s QoL was obtained by PEDQOL if patients where ≤18 years old at the time of study. Health-related QoL is obtained through the PEDQOL questionnaire by seven domains (autonomy, emotional stability, body image, cognition, physical function, and social functionality in family and among friends). An assessment yielding a higher score (self or by proxy) is equivalent to a worse health-related QoL (20).

### Functional capacity questionnaire

With the German daily life ability scale (Fertigkeitenskala Münster-Heidelberg (FMH)), the functional capacity as a measure of QoL was analyzed 1, 3, and 5 years after CP diagnosis/RT (21). FMH uses 56 items, representing the capacity for routine actions in daily life. The test was normalized with 971 persons (45% female participants, aged 0–102 years), resulting in age-dependent percentiles. It takes an average of 4.5 min to answer the FMH questionnaire for first-time users (21).

### Statistical methods

Statistical analysis was performed using SPSS, version 27 (SPSS, Inc.). Data are displayed as median (range) or frequency (percent). Event-free survival (EFS) (OS) rates were assessed using the Kaplan–Meier method. For comparison of continuous variables between two independent groups, the Mann–Whitney *U* test was used. For comparison of different groups for categorical variables, the Fisher’s exact test was used. The Kruskal–Wallis test was used for comparisons of multiple independent samples. EFS was defined as the date of irradiation (PBT or XRT group), diagnosis to the occurrence of an event, or last contact specified for censoring. An event was defined as a >25% increase of residual tumor volume, tumor recurrence after complete surgical resection on central imaging review, or death. OS was defined as date of diagnosis to death of any cause or last contact specified by censoring.

## Results

**Figure 1:**
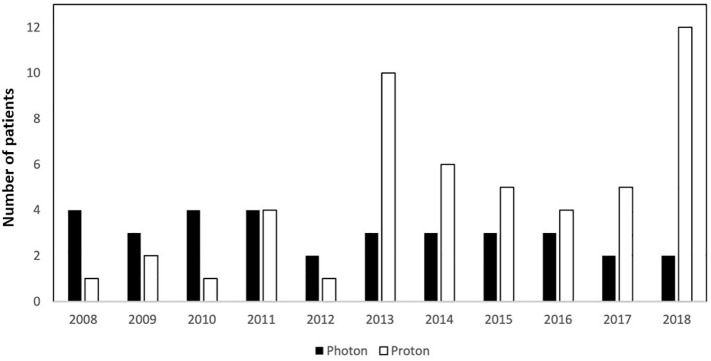
Number of patients treated with proton beam therapy (PBT, white column) or photon-based radiation therapy (XRT, black column) in relation to the year of enrollment in KRANIOPHARYNGEOM 2007.

Between 2007 and 2019, 290 patients with childhood-onset CP were recruited in the trial KRANIOPHARYNGEOM 2007. Of 290 patients (34%), 99 received external RT after diagnosis of adamantinomatous CP, which was confirmed by histological reference assessment in all cases. Of these 99 patients, 64 were treated by PBT (65%), and 35 received XRT (35%). PBT became the predominant irradiation technique during the study period (used in 23% and 77% of all irradiated patients registered within the first and second halves of the recruitment period, respectively) (**Figure 1**). PBT was applied in six different centers treating a median of 6 (range, 2–32) patients with CP, while XRT was applied in 27 different centers treating a median of 1 (range, 1–3) patient with CP. The radiation techniques applied reflect the spectrum commonly used within the follow-up period of the KRANIOPHARYNGEOM 2007 trial. For PBT pencil beam scanning (75%), and for XRT, IMRT (51%) was predominant (**Table 1**).

**Table 1:** Irradiation techniques and integral dose to organs at risk: comparing proton beam therapy with photon-based radiation therapy.

	Proton beam therapy (*n* = 64)	Photon-based irradiation (*n* = 35)
Technique (%)	Passive scatter (5)	3-dimensional conformal radiotherapy (20)
	Uniform scanning (9)	Intensity-modulated radiotherapy (51)
	Pencil beam scanning (75)	Stereotactic radiotherapy (23)
	Unknown (11)	Unknown (6)
Radiation dose to the hypothalamic-pituitary axis in Gray		
Hypothalamus (median, range)		
Minimum	52.6 (0.3–54.5)	51.7 (0.5–54.6)
Mean	53.9 (4.3–55.2)	53.6 (1.4–54.9)
Maximum	54.4 (23.9–56.1)	54.8 (4.1–57.1)
Missing (*n* (%))	16 (25)	16 (46)
Pituitary gland (median, range)		
Minimum	53.4 (47.3–55.0)	51.9 (38.9–54.3)
Mean	54.1 (50.1–55.3)	53.9 (46.1–54.6)
Maximum	54.6 (40.9–56.5)	54.4 (51.4–57.6)
Missing (*n* (%))	22 (34)	15 (43)

Irradiated patients presented with larger (*p* < 0.001) initial tumor volume (PBT: 22.1 cm^3^, range: 0.1–286.3 cm^3^; XRT: 23.0 cm^3^, range: 0.9–107.2 cm^3^) when compared with nonirradiated patients (12.6 cm^3^, range: 0.1–168.7 cm^3^) and a higher rate (*p* = 0.004) of initial hydrocephalus (PBT: 51%; XRT: 52%; non-RT: 32%). However, differences in terms of presurgical HI and HL as potential risk factors for the development of a hypothalamic syndrome were nondetectable between patients with PBT, patients with XRT, and patients without RT (**Table 2**). Complete surgical resections were more frequently (*p* = 0.001) achieved in nonirradiated patients (30%) when compared with patients treated with PBT (17%) and XRT-treated patients (3%).

**Table 2:** Characteristics of the study population showing data for patients with childhood-onset adamantinomatous craniopharyngioma (CP) recruited in KRANIOPHARYNGEOM 2007 treated with photon-based radiation therapy (XRT), proton beam therapy (PBT), and patients without external RT.

	XRT	PBT	Irradiated patients	Nonirradiated patients	*p*-value^*^
***n***	35	64	99	123	
**Sex (male/female, *n* (%))**	15 (43)/20 (57)	34 (53)/30 (47)	49 (49)/50 (51)	59 (48)/64 (52)	
**Age at CP diagnosis (years, median (range))**	9.2 (2.3–16.6)	9.8 (1.6–17.9)	9.5 (1.6–17.9)	8.8 (1.3–21.3)	
**Follow-up interval (years, median (range))**	8.0 (2.4–14.7)	5.2 (0.9–14.1)	6.4 (0.9–14.7)	6.1 (0.1–14.0)	
**Age at irradiation (XRT) (years, median (range))**	10.0 (5.6–17.2)	10.9 (2.5–20.8)	10.5 (2.5–20.8)	–	
**Interval event–XRT (years, median (range))**	0.2 (0.1–4.5)	0.2 (0.1–3.5)	0.2 (0.1–4.5)	–	
**Interval CP diagnosis–event (years, median (range))**	0.8 (0.1–14.2)	1.0 (0.2–5.6)	1.0 (0.01–14.2)	–	
**XRT dose, (Gray; median (range))**	54.0 (50.4–54.0)	54.0 (50.4–54.0)	54.0 (50.4–54.0)	–	
**Hydrocephalus**	18 (51)	33 (52)	51 (51)	40 (32)	0.004
**Tumor size (cm³, median (range))**	23.0 (0.9–107.2)	22.1 (0.1–286.3)	22.7 (0.4–286.3)	12.6 (0.1–168.7)	<0.001
Reason for XRT					
**Event (relapse, progression)**	21 (60)	51 (80)	72 (73)	–	
**Residual tumor**	12 (34)	6 (9)	18 (18)	–	
Degree of surgical resection					
**Complete resection**	1 (3)	10 (17)	11 (11)	37 (30)	0.001
**Incomplete resection**	34 (97)	49 (77)	83 (83)	84 (68)	
Hypothalamic involvement (HI) (17)					
**Grade 0 (no HI)**	2 (6)	2 (3)	4 (4)	8 (6)	0.057
**Grade 1 (anterior HI)**	6 (17)	10 (16)	16 (16)	35 (28)	
**Grade 2 (ant. + posterior HI)**	27 (77)	51 (80)	78 (79)	80 (65)	
Hypothalamic lesion (HL) (17)					
**Grade 0 (no HL)**	13 (37)	15 (23)	28 (28)	34 (28)	
**Grade 1 (anterior HL)**	13 (37)	25 (39)	38 (38)	41 (33)	
**Grade 2 (ant. + posterior HL)**	9 (26)	23 (36)	32 (32)	48 (39)	
Endocrine deficits at last contact (*n* (%))					
**Diabetes insipidus**	24 (69)	58 (83)	77 (78)	95 (77)	
**Hypothyroidism**	30 (86)	59 (92)	89 (90)	101 (82)	
**Hypocortisolism**	29 (83)	56 (87)	85 (86)	101 (82)	
**Hypogonadism**	16 (46)	27 (42)	43 (43)	53 (43)	
**Growth hormone deficiency**	27 (77)	43 (67)	70 (71)	78 (63)	

^*^p-values refer to the comparison of irradiated and nonirradiated patients. For comparison of continuous variables between two independent groups, the Mann–Whitney U test was used. For comparison of different groups for categorical variables, the Fisher’s exact test was used.

External RT was started at a median interval of 11 months (range: 1–70 months) after CP diagnosis, whereas PBT was started at a median interval of 13 months (range: 1–70 months) and XRT at a median interval of 7 months (range: 3–63 months) after CP diagnosis. Reasons for RT (PBT/XRT) were preceding events in terms of relapses after complete resection (14%/3%) and progression of residual tumor after incomplete resection (59%/57%), respectively. Residual CP after incomplete surgical resection without signs of progression was the reason for RT in 18 patients (9% PBT; 34% XRT). The doses (Gray (Gy)) of radiation therapy were similar for PBT (median: 54.0 Gy; range: 50.4–54.0 Gy) and XRT (median: 54.0 Gy; range: 50.4–54.0 Gy). Dosismetric evaluations of organs at risk were available in 52 of 64 (81%) patients treated with PBT and 30 of 35 (86%) patients treated with XRT. Within the patients with dosimetric evaluation, hypothalamus and pituitary were contoured in the XRT group at 69% and 72%, respectively, and in the PBT group at 92% and 81%, respectively. The doses to the hypothalamic-pituitary axis were very similar when comparing PBT and XRT (**Table 1**).

**Figure 2:**
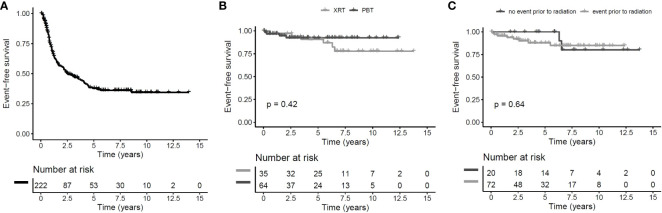
Event-free survival (EFS) of childhood-onset craniopharyngioma (CP) patients recruited between 2007 and 2019 in KRANIOPHARYNGEOM 2007. Cohort of all patients with EFS defined as time from diagnosis to first event and censoring at end of follow-up or in case of irradiation without prior event **(A)** patients receiving proton beam therapy (PBT) compared to patients receiving photon-based irradiation since the start of radiotherapy with log-rank test with EFS defined as time from irradiation to first event after start of irradiation **(B)** and patients receiving irradiation after a prior event compared to patients receiving irradiation without an event since the start of radiotherapy with log-rank test with EFS defined as time from irradiation to first event after start of irradiation **(C)**.

The overall follow-up interval of surviving patients was 6.4 years (range: 0.9–14.6 years) in irradiated patients and 6.1 years (0.1–14.0 years) in nonirradiated patients. The 5-year EFS rate for the whole group of CP patients (*n* = 222) was 38% ± 4% (**Figure 2A**). The EFS after irradiation was higher, while there was no clear difference between PBT- (*n* = 64) and XRT-treated (*n* = 35) patients (PBT: 5-year EFS rate: 92% ± 4% vs. XRT: 91% ± 5%, *p* = 0.42) (**Figure 2B**). Whether prior to radiation an event (*n* = 20) or no event (*n* = 72) occurred did not influence EFS (5-year EFS rate 100% ± 0% vs. 88% ± 4%, *p* = 0.64) (**Figure 2C**). OS rates were similar in all analyzed subgroups. Only one fatal event occurred in the PBT subgroup. The patient had a long course of disease with a lot of complications, including cerebral infarction, but the reason of death was not declared.

No irradiation-related secondary malignancies were observed in any of the three subgroups.

**Figure 3:**
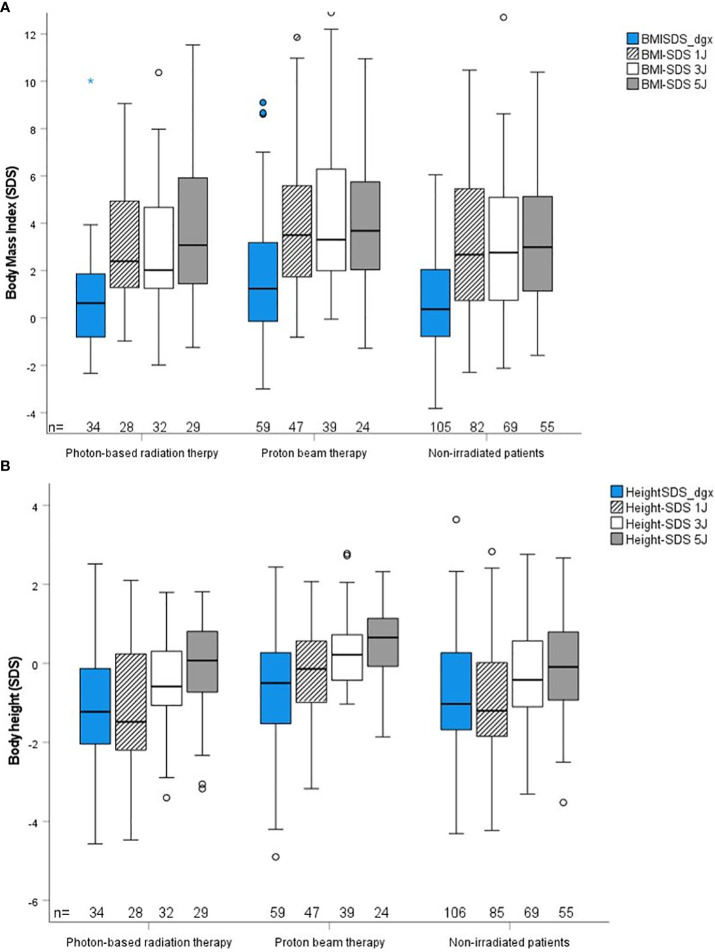
Anthropometric parameter body mass index (BMI) SDS (19) **(A)** and body height SDS (18) **(B)** during follow-up in childhood-onset craniopharyngioma (CP) patients (recruited in KRANIOPHARYNGEOM 2007) treated with photon-based radiation therapy (XRT), with proton beam therapy (PBT), and in CP patients without external irradiation at defined time points. Blue boxes: time point at diagnosis; crossed lines boxes: time point 1 year after diagnosis/RT; white boxes: time point 3 years after diagnosis/RT; grey boxes: time point 5 years after diagnosis;RT. The horizontal line in the middle of the box depicts the median. The top and bottom edges of the box, respectively, mark the 25th and 75th percentiles. Whiskers indicate the range of values that fall within 1.5 box lengths. The Kruskal–Wallis test was used for comparisons of multiple independent samples.

**Figure 4:**
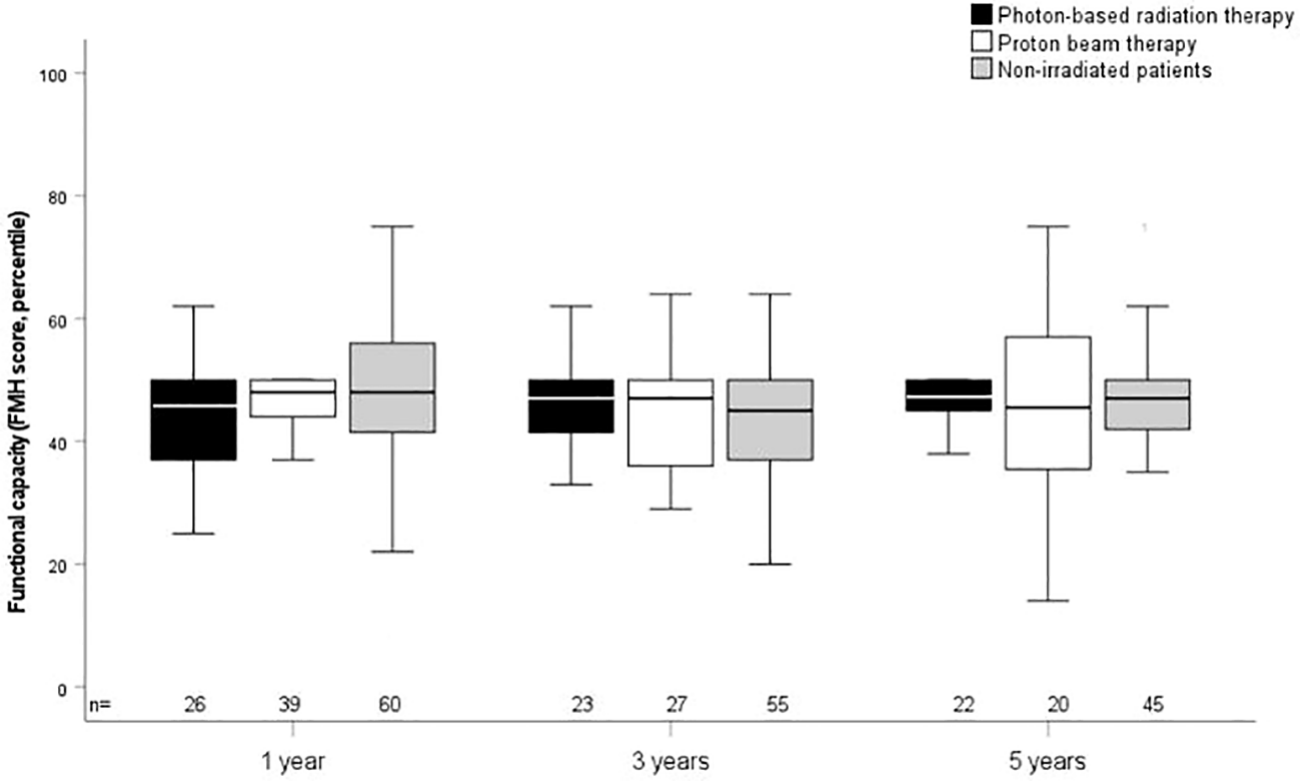
Functional capacity as measured by the capability scale Fertigkeitenskala Münster Heidelberg (FMH) in childhood-onset craniopharyngioma (CP) patients (recruited in KRANIOPHARYNGEOM 2007) treated with photon-based radiation therapy (XRT), with proton beam therapy (PBT), and in CP patients without external irradiation at the defined time points. White boxes: PBT; black boxes: XRT; grey boxes: no external irradiation. FMH scores are shown for the time points 1 year, 3 years, and 5 years after irradiation/CP diagnosis. The horizontal line in the middle of the box depicts the median. The top and bottom edges of the box, respectively, mark the 25th and 75th percentiles. Whiskers indicate the range of values that fall within 1.5 box lengths.

**Figure 5:**
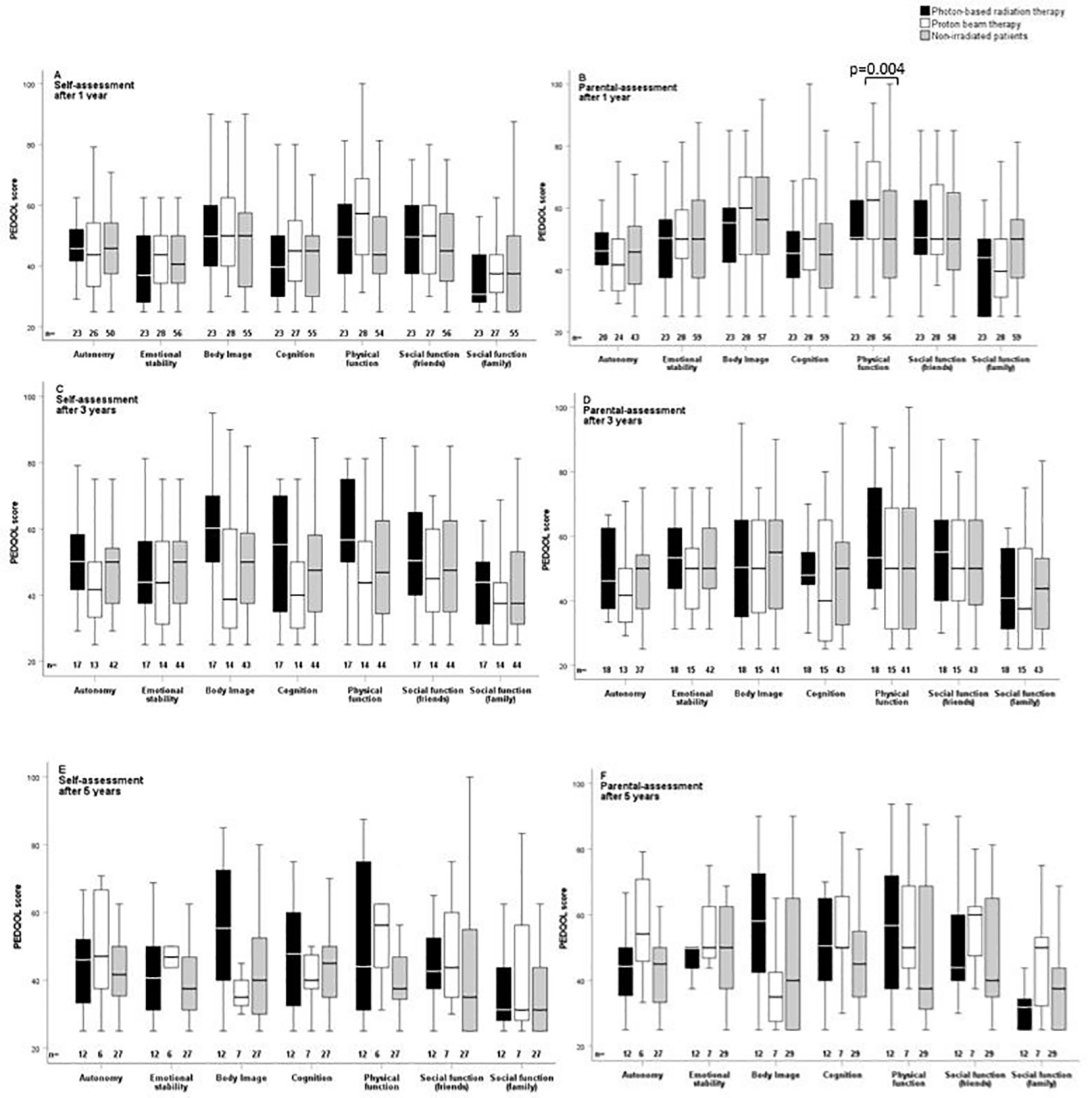
Self-assessment **(A, C, E)** and parental assessment **(B, D, F)** of quality of life by the Pediatric Quality of Life questionnaire (PEDQOL) in childhood-onset craniopharyngioma (CP) patients recruited in KRANIOPHARYNGEOM 2007 with regard to external irradiation. White boxes: proton beam therapy (PBT); black boxes: photon-based radiation therapy (XRT); grey boxes: no external irradiation. PEDQOL scores are shown as negative ratings at the time points 1 year **(A, B)**, 3 years **(C, D)**, and 5 years **(E, F)** after RT/CP diagnosis. The horizontal line in the middle of the box depicts the median. The top and bottom edges of the box respectively mark the 25th and 75th percentiles. Whiskers indicate the range of values that fall within 1.5 box lengths.

The outcome parameters—endocrine status, anthropometric measurements, functional capacity, and QoL—were assessed at defined timepoints (1, 3, and 5 years after CP diagnosis/RT). Of all tested patients at their last visit before RT, 84% (*n* = 63) had diabetes insipidus, 87% (*n* = 68) hypothyroidism, 61% (*n* = 61) had growth hormone deficiency (GHD), 84% (*n* = 61) had hypocortisolism, and 35% (*n* = 15) had hypogonadism. Analyzing all patients at their last visit after irradiation with regard to endocrine status revealed no significant difference between PBT- and XRT-treated or irradiated and nonirradiated patients (**Table 2**). Patients with PBT, XRT, and without RT presented with similar BMI SDS and height SDS during follow-up (**Figure 3**). Differences in terms of functional capacity were not observed between patients after PBT or XRT and patients without RT during follow-up (**Figure 4**). In terms of QoL, only for the PEDQOL domain physical function, parental-assessed QoL was lower 12 months after PBT compared with CP patients after XRT and patients without RT (**Figure 5**).

## Discussion

In our study, we compared the outcome between 99 irradiated CP patients and 123 nonirradiated CP patients, proving that hypothalamic sparing surgery and irradiation, irrespective of the technique used, are efficient treatments with 5-year EFS rates over 90%. This approach has been at least equivalent in maintaining “historical cure rates” by more aggressive surgery while not increasing morbidity. A surgical cure is only advisable in smaller tumors. In our cohort, baseline tumor differences in terms of hydrocephalus and tumor volumes already existed in those receiving any form of irradiation. They predispose these patients *a priori* before radiation, and not as a result of it, to sequelae (10, 22, 23).

Risk-appropriate treatment of CP is a challenging multidisciplinary task (8, 11, 24). Based on recent reports, hypothalamus-sparing surgical strategies are preferred in childhood-onset CP patients presenting initially with a sellar mass involving hypothalamic structures (8, 10, 25). Accordingly, radiooncological approaches for residual tumor treatment after incomplete hypothalamus-sparing surgical interventions play a major role in the current consensus on appropriate treatment for CP patients (11, 26). The occurrence of disease progression or not before irradiation was not associated with a difference in EFS. Therefore, it seems to be better to postpone irradiation of CP patients with residual tumor until the first progression.

Modern, highly conformal, intensity-modulated RT techniques are especially advantageous in treating pediatric CP patients. The tumor in general is well demarcated toward healthy tissue and does much less infiltrate it compared to other brain tumors. In patients irradiated according to the KRANIOPHARYNGEOM 2007 trial, the target volume included all relevant tissues in contact with previous tumor (tumor bed of the primary tumor, tumor bed of relapse/progressive tumors, and residual tumor), regardless of the intervening time interval. In cases of very extensive disease, compromises to reduce the target volume were performed, depending on an individual risk assessment. To reduce the irradiation volume and associated increased probabilities of toxicities, in more recent guidelines like that for the KRANIOPHARYNGEOM Registry 2019, areas with an expected low risk of relapse (e.g., areas of cysts with resected cyst walls, tumor beds of primary tumors in cases of late relapses) could be excluded, although we herein show that most endocrinopathies were not caused by irradiation.

PBT is increasingly applied in pediatric central nervous tumors, and there is a consensus among treating physicians that it is the treatment of choice in CP patients (27, 28). PBT patterns of care studies often do not record the utilization of both PBT and XRT) (29). In our study, we compared the PBT with XRT patterns of care in a prospective trial with population-based recruitment in place. Over the inclusion period of 12 years, PBT became the predominant irradiation technique. PBT was performed centralized in only a few facilities, while XRT was applied in many different facilities, treating in median only one patient per recruitment period of 12 years. However, whether a more centralized RT increases treatment quality needs to be determined in the future.

We further compared unpaired data from 64 PBT versus 34 XRT patients, testing the hypothesis that one RT technique (PBT) causes less hypothalamic obesity, endocrinopathy, and functional morbidity than the other by reducing scatter to critical organs at risk, which include the HPA and optic pathway, without compromising cure.

The conventional cumulative target dose (total volume dose) applied in CP is 54.0 Gray (13, 30–36). After XRT, we observed a 5-year EFS rate of 94% that is comparable to 10- and 20-year PFS rates of 95% and 54%, respectively, reported before in the literature (37–43). Using PBT, the 5-year EFS rate was 92% in our cohort and in line with earlier reports of 5- and 10-year local control rates of 93% and 85%, respectively, and a 10-year OS rate of 72% (44). Our results demonstrate that PBT is not compromising the cure compared with XRT.

The HPA includes critical structures that are often disturbed in CP patients (11). Our study demonstrates that most HPA deficits occur before RT due to CP or the surgery performed before the irradiation, with diabetes insipidus, hypothyroidism, hypocortisolism being more common than GHD, and hypogonadism. As shown by height SDS decrements, GHD is often already clinically existent before diagnosis of CP, and laboratory testing is suspectable to faults (6). This can also be seen in **Figure 3B**, but a longitudinal intergroup comparison of BMI or height SDS was not within the scope of this study. Because of the already preexistent damage, a clear benefit of PBT compared to XRT with regard to HPA deficits could not be expected. Studies on patients with suprasellar optic gliomas confirm that endocrinopathies are more tumor- or surgery-induced than radiation-induced (45, 46).

For multicenter trials, it is a challenge to diagnose endocrinopathies uniformly and receive robust data. However, this challenge needs to be overcome in future trials or registries in order to establish endocrine event-free survival (EEFS) and endocrine morbidity score (EMS) as important outcome parameter together with QoL and functional capacity (45, 46). In the PEDQOL domain “physical function”, parental-assessed QoL was lower 12 months after PBT versus XRT or non-RT patients but not again at the other time points, so the clinical relevance remains inconclusive. In addition, differences in our CP cohort in terms of anthropometric parameters, functional capacity, and health-related QoL as outcome parameters were not observed between different RT techniques. These results confirm previous reports and trials performed in smaller cohorts with shorter follow-up intervals (3, 5, 6, 24, 47, 48).

It has been shown before that the outcome parameters depend on the degree of HL, especially those involving posterior hypothalamic structures (8, 11). For that reason, radical surgery in this region should be avoided, and RT should be applied. A dosimetric comparison of treatment plans in our cohort demonstrated that there were no relevant differences in radiation doses to hypothalamus or pituitary gland between PBT and XRT. The hypothalamus or HPA, respectively, are so close to residual CP and within or near the clinical target volume that both structures likely receive a full dose irrespective of whether XRT or PBT was applied (49). Of note, not all patients with dosimetric evaluations received a contouring of the hypothalamus and pituitary gland, probably due to a displacement by the CP or surgery.

A dosimetric comparison between PBT and XRT treatment plans for CP demonstrates that PBT is associated with a lower dose to important structures like the hippocampus, subventricular zone, and the vascular system (15). Initial publications of first-generation PBT suggested that, in comparison to XRT, the rate and pattern of treatment failure and occurrence rates of vasculopathy, necrosis, and severe neurological and neuroendocrine sequelae were equivalent (27). Due to the anatomical location of CP close to the Circle of Willis, the rate of vasculopathy after RT was 5%–32% (34, 47, 50–53). Many of these vasculopathies have only been detected on MR angiography and are subclinical. It has been shown that, besides RT, the tumor or surgical manipulation contributes predominately to the development of vasculopathies (54). In line with these results, we have shown within the cohort of KRANIOPHARYNGEOM 2007 patients that all cerebral infarctions (11%, 28 of 244 patients) were detected on MRI before RT and mainly after surgery (55). However, the use of MRI angiography was not established for follow-up in the KRANIOPHARYNGEOM 2007 cohort, so the incidence of subclinical vasculopathies may be underreported and especially no comparison between PBT and XRT can be drawn yet.

It has been shown in a direct comparison with a correction for the distribution of RT dose to a normal brain that patients treated with PBT had no change in academic (i.e., reading and math) achievement scores while those receiving XRT demonstrated a significant decline (56). While these preliminary reports are promising, we did not observe a difference in functional capacity and health-related QoL in the KRANIOPHARYNGEOM 2007 cohort that would be in accordance with these results.

However, in future studies it is important to analyze the locoregional distribution of radiation doses to normal brain tissue beyond the HPA, comparing PBT and XRT in a large cohort like that of the KRANIOPHARYNGEOM 2007 trial (15). The results need to be correlated to neurocognitive function in order to receive an idea of the brain damage caused by different irradiation techniques (57).

The results of our study are limited, and some observations are speculative at this point. Due to the rareness of the disease, the cohort size of our study remains improvable. Nevertheless, we are reporting on the outcome of the—to our knowledge—largest group of childhood-onset CP patients treated with different radiooncological techniques. With that focus, no comparisons of the outcome parameters to healthy controls were obtained. Furthermore, our investigation was done retrospectively and not in a randomized fashion. The utilization of both radiation techniques was different over time, with PBT being more predominant to the end of the inclusion period, so that a bias through other evolving techniques in XRT cannot be excluded. The follow-up period after PBT is still short in our study. However, major clinical manifestations of severe side effects such as hypothalamic syndrome or consecutive severe hypothalamic obesity have already been observed after short-term follow-up, i.e., during the first year after surgery (5, 6, 8). Increasing patient numbers and prolonging follow-up for assessment of long-term outcomes are goals of future studies on CP survivors.

We conclude that PBT is similarly efficient in preventing relapses and progressions in childhood-onset CP patients when compared with patients treated with XRT. During follow-up, clinically relevant differences between PBT and XRT in terms of QoL, functional capacity, and the degree of obesity as a marker of hypothalamic syndrome were not observed. Studies on larger CP cohorts with longer follow-up after RT are warranted in order to analyze whether PBT has the advantage of preventing long-term sequelae such as second malignant neoplasms, neurocognitive deficits, and vasculopathies.

## Data availability statement

The raw data supporting the conclusions of this article will be made available by the authors, without undue reservation.

## Ethics statement

The studies involving humans were approved by local standing-committee on ethical practice of the Medizinische Fakultät, Julius-Maximilians-Universität Würzburg, Germany (140/99; 94/06, respectively). The studies were conducted in accordance with the local legislation and institutional requirements. Written informed consent for participation in this study was provided by the participants’ legal guardians/next of kin.

## Author contributions

CF researched the data and wrote the manuscript. SB collected data and prepared statistical analyses, contributed to the analytical plan and discussion and reviewed/edited the manuscript. MB collected data and reviewed/edited the manuscript. JB contributed to the analytical plan and discussion and reviewed/edited the manuscript. PS contributed to the discussion and reviewed/edited the manuscript. GC contributed to the discussion and reviewed/edited the manuscript. ME performed statistical analyses, contributed to the analytical plan and discussion and reviewed/edited the manuscript. CV collected data and reviewed/edited the manuscript. BB did neuroradiological assessment of all imaging. BB is the neuroradiologist, who performs reference-assessment of imaging in all patients recruited in KRANIOPHARYNGEOM 2007. She prepared the imaging data and their presentation and reviewed/edited the manuscript. SH collected data and reviewed/edited the manuscript. BT did a radiooncological assessment in all cases. BT is the radiooncologist who performs reference assessment of all patients recruited in KRANIOPHARYNGEOM 2007. HM initiated and conducted the multicenter trial KRANIOPHARYNGEOM 2007, contributed to the analytical plan and discussion, and reviewed/edited the manuscript. All authors contributed to the article and approved the submitted version.
